# Prevalence of inappropriate antibiotic doses among pediatric patients of inpatient, outpatient, and emergency care units in Bangladesh: A cross-sectional study

**DOI:** 10.1371/journal.pgph.0003657

**Published:** 2024-09-10

**Authors:** A. F. M. Mahmudul Islam, Md. Abu Raihan, Khandaker Tanveer Ahmed, Md. Saiful Islam, Nahria Amin Nusrat, Md. Asif Hasan, Md. Galib Ishraq Emran, Ananta Kumar Das, Anika Bushra Lamisa, Tania Ahmed, Halima Akter Happy, Mst. Mahfuza Khatoon

**Affiliations:** 1 Department of Pharmacy, Gono Bishwabidyalay, Savar, Dhaka, Bangladesh; 2 Department of Microbiology, Jahangirnagar University, Savar, Dhaka, Bangladesh; 3 Department of Statistics, Jahangirnagar University, Savar, Dhaka, Bangladesh; 4 Department of Public Health & Informatics, Jahangirnagar University, Savar, Dhaka, Bangladesh; 5 Department of Pharmaceutical Sciences, School of Health and Life Science, North South University, Dhaka, Bangladesh; 6 Department of Environmental Sciences, Jahangirnagar University, Savar, Dhaka, Bangladesh; 7 Department of Biochemistry and Microbiology, School of Health and Life Science, North South University, Dhaka, Bangladesh; Eduardo Mondlane University: Universidade Eduardo Mondlane, MOZAMBIQUE

## Abstract

The heterogeneous pediatric populations, their physiological differences, along with the necessity of performing additional dose calculation, make the pediatric population more vulnerable to the incidences of inappropriate antibiotic doses. This study was conducted to examine and evaluate the appropriateness of antibiotic doses. A cross-sectional study with a quantitative approach was conducted in three hospitals located in Savar from January 06, 2021 to October 17, 2022. This study had used a convenient sampling method to collect 405 filled prescription orders from heterogeneous pediatric patients prescribed by physicians from emergency, inpatient, and outpatient care units of various clinical settings. The Harriet Lane Handbook was used as reference to investigate inappropriate doses of antibiotics. Subsequently, all analyses were conducted using the RStudio 1.3.959 software. Binary logistic regression was used to assess the risk of inappropriate antibiotic prescription in pediatrics. The overall prevalence of inappropriate antibiotic dosing in pediatrics was 335 out of 545 (61.5%). Overdosing (36.3%) and oral antibiotic prescriptions (64%) were more common than underdosing (20.4%) and parenteral antibiotics (36%). The majority (230 out of 405, 56.8%) of pediatric patients had prescriptions with inappropriate antibiotic doses, with prevalence rates of 33.8% for inpatients, 86.7% for outpatients, and 50% for emergency pediatrics. The results also indicated that pediatric patients in outdoor and emergency care units, infants, toddlers, and early childhood, those prescribed two antibiotics simultaneously, and those receiving parenteral antibiotics, were less likely to have inappropriate antibiotic dosages in their prescriptions. This study demonstrated that about one out of every two prescriptions had inappropriate antibiotic doses; in particular, prescriptions containing only one antibiotic exhibited a substantial proportion of inappropriate antibiotic doses. Inappropriate antibiotic doses may result in therapeutic failure, patient harm, and antibiotic resistance. Good clinical pharmacy practice and careful adherence to pediatric dosing standards may minimize inappropriate antibiotic doses.

## Introduction

Antibiotics are the most promising medications prescribed for treating bacterial infections, and frequently prescribed to pediatric patients for various infectious diseases [1]. Studies have reported that at least half of the pediatric patients received antibiotic during their treatment in the hospital [2–5]. However, the pediatric population is not homogeneous in nature. Rather, they are classified based on their age into distinct categories, which are neonatal, infancy, toddler, early childhood, middle childhood, early adolescence, and late adolescence [6]. Specifically, multiple steps are required to follow in using any medication in pediatric patients that make it a complicated and error-prone process where accurate weight and age considering dose calculation, verification, and administration of doses have to be strictly followed [7]. Furthermore, there is little or no instruction available on how to adjust a child’s dosage [8] Moreover, multiple studies have been confirmed that inappropriate antibiotic dosage selection is one of the top-ranked inappropriate antibiotic prescribing in pediatric patients [9–12]. The term "inappropriate antibiotic prescribing" (IAP) refers to the inappropriate utilization of antibiotics as well as the improper selection, dose, or course of treatment [4, 11, 13]. Currently, the most common medication errors in pediatric patients are inappropriate dosing errors [14–16]. Morbidity and mortality due to medication errors are the 14th leading causes in the world [17]. Additionally, a study reported that about 6.5% morbidity and mortality in pediatric inpatients is due to inappropriate antibiotic prescription [18]. In Bangladesh, there is no such official data of morbidity and mortality in pediatric inpatients due to medication errors.

Evidence suggested that 30% to 50% inappropriate antibiotic prescribing happened in the United States and Canada [19, 20]. Consequently, inappropriate antibiotic use was found at a rate of 47% in Turkey, 41% in Pakistan, and 28% in Ethiopia [10, 11, 13]. Although Standard Treatment Guidelines (STG) on Antibiotic Use in Common Infectious Diseases of Bangladesh [21] are readily available and public healthcare is accessible, the country still faces issues of antibiotic overuse, similar to other low- and middle-income countries (LMICs) [22, 23]. However, a recent global study revealed a significant increase of 35% in the usage of antibiotics in low- and middle-income countries (LMICs) [24]. An appropriate prescription culture is a result of the disparity between theoretical and clinical approaches to prescribing [25], doctors’ anxiety about losing patients [26], pharmaceutical industry-sponsored information, and product-biased prescribing practices [27, 28]. Research conducted in Bangladesh revealed that approximately 83% of antibiotic prescriptions in the country are issued without prior clinical assessment [29].

The available antibiotic dosages in the market are designed for adults. Therefore, to ascertain the appropriate dosage for a pediatric patient based on their age and weight, further dosage calculations and manipulation of the available dosages are required. However, numeracy errors such as 10-fold error and incorrect dosage calculations can have major negative impacts on patients [30]. As a result, medication errors in pediatric patients can cause harm three times more than in adults [31]. In addition, multiple studies confirmed that inappropriate antibiotic prescriptions can lead to antibiotic resistance, ineffective first-line antibiotic treatment, infection recurrences, and even death, especially when dosages are low and administered over extended periods [32–34]. Therefore, prescribing antibiotics at the recommended doses to pediatric patients ensures their safety and efficacy and reduces the incidence of inappropriate antibiotic prescriptions. The principles of rational antibiotic use are well-defined, and inappropriate prescribing patterns are reported for adults, however there is insufficient data regarding pediatric patients’ dose errors. The study was designed to quantify the prevalence of inappropriate antibiotic dosing in pediatric patients within inpatient, outpatient, and emergency care units in Bangladesh. The study will also investigate the association between incorrect dosing and the use of oral or parenteral antibiotics, as well as the impacts of prescribing multiple antibiotics. Additionally, the research aimed to identify the proportion of dosing errors that are attributable to overdoses and underdoses, while also determining which age groups of pediatric patients are most susceptible to inappropriate antibiotic doses.

## Materials and methods

### Study design, population and setting

A research study was conducted with pediatric patients in three distinct care units: inpatient, outpatient, and emergency care. The study was conducted in three hospitals situated in Savar, Bangladesh, spanning from January 6, 2021, to October 17, 2022. Throughout this time frame, we documented prescriptions as well as relevant information provided by caregivers. The inclusion criteria of this study included pediatric patients who were below the age of 14 years, presented with mild to moderate infections, and were receiving care across various settings, including inpatient, outpatient, and emergency care units, with a prescription for antibiotics. The exclusion criteria included pediatric patients who were undergoing treatment with medications other than antibiotics, those who were unable to participate due to acute or chronic clinical conditions, and pediatric patients with abnormal kidney or liver functions.

### Sampling method and recruiting process

Non-probability sampling was used here due to the lack of a sampling frame of compatible patients. Convenient sampling, selecting participants based on accessibility and availability, is the most common method in clinical and quantitative research [35, 36]. Face-to-face interviews, employing pre-designed printed questionnaires, were conducted with caregivers or parents of pediatric patients. Children were included only if their parents or caregivers were actively involved in or knowledgeable about their treatment.

### Study area & sample size

**Fig 1:**
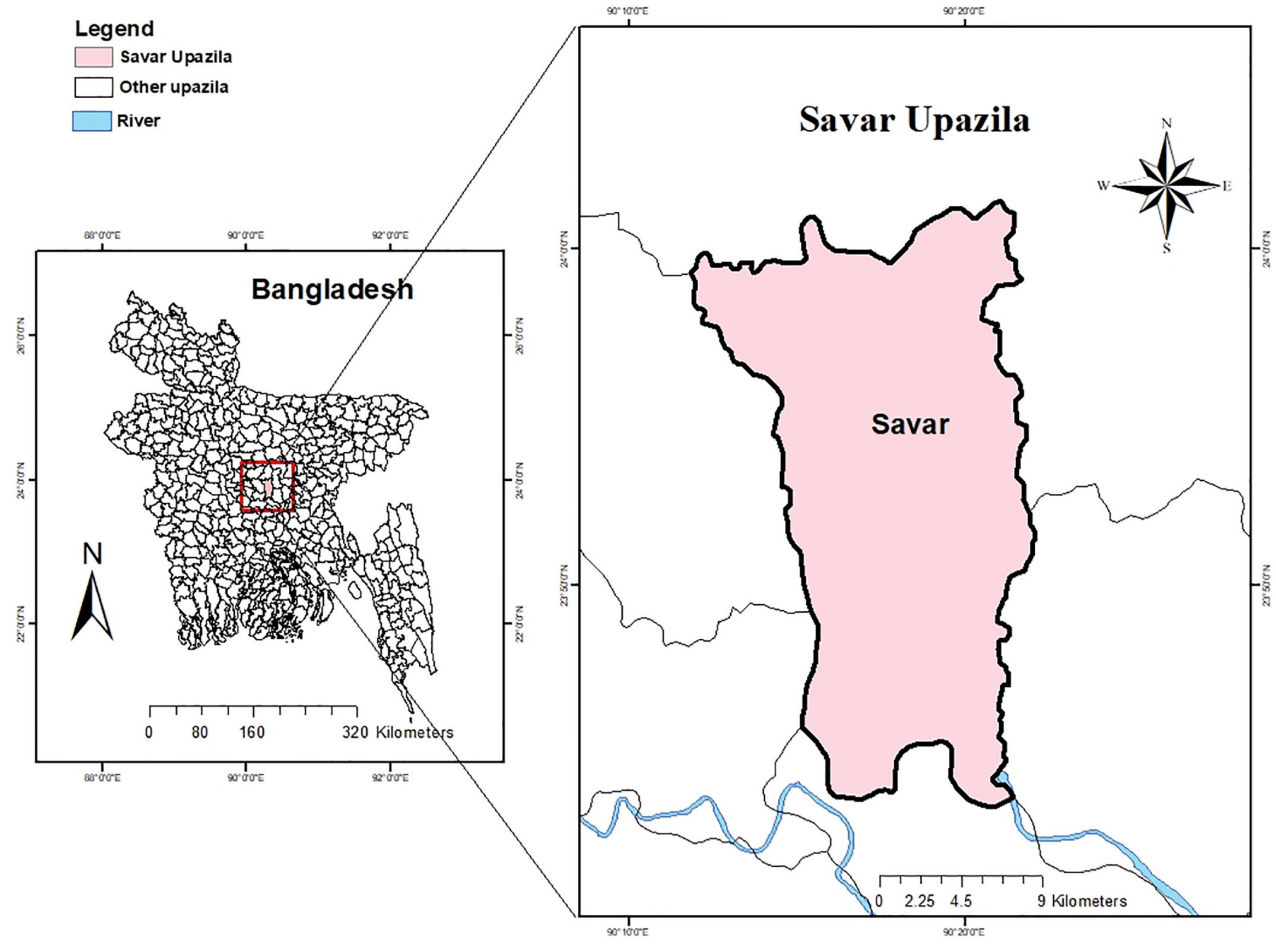
A map illustrating the geographical distribution of pediatric patients at Savar in Bangladesh. The map was generated by utilizing a shapefile obtained from the publicly available GADM database [37].

Our investigation relied on the Savar region. Patients who fulfilled the prerequisites and expressed their willingness to disclose prescription information were chosen from three hospitals. The survey area is illustrated in Fig 1, which depicts the distribution of pediatric patients in Savar, Bangladesh, from a geographical perspective.

When determining the appropriate sample size, we took into account practical constraints, research questions, and the level of precision desired. We selected 405 antibiotic-prescribed pediatric patients from a total of 714 patients in hospital care units during the study period as study samples using convenient sampling.

### Ethics

All procedures of the present study were carried out according to the principle of human investigation by the Institutional Research Ethics Committee. Formal ethical approval was granted by the ethical review board of Gono Bishwabidyalay, Savar, Dhaka, Bangladesh (Ref. no: CMR/EC/001). The objectives and procedures of the research study were informed to all participants. Before enrollment, the adolescents (aged above 12 years) provided verbal assent, which was then recorded in a formal document. The parental or legal guardian’s written consent was subsequently obtained. In situations involving adolescents who were incapable of providing verbal consent on account of illness, consent was exclusively obtained from their parent or legal guardian. In such instances, proxy data were collected. However, fingerprints were utilized as a means of identification for parents or legal guardians who were incapable of providing a signature or writing their names. Furthermore, another person was serving as an impartial witness, also signed the Informed Consent Form (ICF) during the investigation to ensure precise communication and validating the consent form as evidence of this. The study prioritized the privacy and confidentiality of personal information by anonymizing the data and employing a coding system for analysis.

### Study instrument

The semi-structured questionnaire (S1 File) was designed to collect information about the characteristics of pediatric patients, as well as the antibiotics they were prescribed, the doses of the antibiotics, and the specific dosage regimens they followed during their treatment. The questionnaire was translated into the Bangla language (participants’ first language) to prevent further errors and facilitate better comprehension and response (S2 File). The questionnaire was divided into two sections. The primary focus of the first section was on the demographic profiles of pediatric patients. Age, gender, and patient type were asked during the study periods. We employed ‘patient type’ to classify patients by treatment environment in our study. ‘Inpatients’ stayed overnight in the hospital for treatment, ‘outpatients’ received treatment without an overnight stay, and ‘emergency patients’ sought quick care in the emergency department.

Age was categorized into five age ranges according to the National Institute of Child Health and Human Development (NICHD) pediatric terminology: term neonatal (birth to 27 days), infancy (28 days to 12 months), toddlers (13 months to 2 years), early childhood (2 to 5 years), middle childhood (6 to 11 years), and adolescent (12 to 18 years). The second section of the study questionnaire focused solely on the medication history to document the list of antibiotics and the clinical complications for which they were prescribed.

### Study procedures

Data was collected using a survey questionnaire consisting of both structured and open-ended questions. This study specifically focused on antibiotic prescriptions for pediatric patients. The appropriateness of dosing was assessed by comparing the prescribed antibiotics to the recommended doses based on the age and weight of the pediatric patients, using the most recent version of the Harriet Lane Handbook as a reference [38]. Antibiotic overdose was assumed when the calculated weight-based daily dose (mg/kg/day) exceeded the Harriet Lane Handbook’s [38] maximum recommended pediatric dose for a specific antibiotic [38]. When the prescribed weight-based daily dose (mg/kg/day) was less than the recommended pediatric dose in the reference book, it was assumed that the antibiotic was administered sub-therapeutically or insufficiently. If any antibiotic dosage was an overdose or underdose, it was assumed that the antibiotic dosage in this study was inappropriate. In our study, a ±10% dosing deviation was considered inappropriate antibiotic doses upon comparing the prescribed dosage to the guideline recommended in Harriet Lane Handbook. In this regard, an antibiotic dosing error or inappropriate antibiotic dosing may be defined as any prescription having daily dosing of 110% or above the maximum recommended daily dose or below 90% of the daily recommended minimum antibiotic doses. The same margin of dosing error was followed in two recent research works by Xavier et al and Iftikhar et al. [11, 34].

### Statistical data analysis

The data collected from the questionnaire underwent a comprehensive processing workflow to ensure accuracy and reliability in our analysis. Initially, the raw data was inputted into Microsoft Excel 2019 for collection purposes. Subsequently, utilizing Excel, the data underwent a meticulous cleansing, editing, and organization process. Once the dataset was refined, it was imported into RStudio 1.3.959 for the statistical analyses. To comprehensively analyze the data, frequency percentages were used for socio-demographic factors and antibiotics. Cross-tabulations explored the relationship between dose levels, administration frequency, patient type, age group, dosage regimen, and dosage form. Significance was determined through Pearson’s Chi-square tests, reporting only relevant dependencies. Deviations from standard dose levels were identified using median absolute deviations, calculated with Kruskal-Wallis tests assessing significance. Univariate and multivariate logistic regression model identified predictor variables for inappropriate antibiotic use. The multivariate model incorporated the following variables: age category, patient weight, patient type, number of prescribed antibiotics, dosage regimen, and dosage form. Crude and adjusted Odds Ratios (OR) estimated the likelihood, with a 95% confidence interval (CI) and p-value <0.05 indicating significance. Multicollinearity analysis ensured the reliability of the regression model by assessing independent variable correlations.

### Participant’s consent

Given that the study involved children, the majority under 14, parental or guardian consent served as authorization. In both Bangla and English, an Informed Consent Form (ICF) was provided, detailing objectives, interview duration, selection criteria, and the signature option for legal guardians. Emphasis was placed on confidentiality. For illiterate guardians, investigators read and explained the form. Handwritten signatures were obtained before interviews, ensuring voluntary participation. Participants shared medical histories. Parents, caregivers, or attendees willingly provided personal information, clinical details, and prescription data related to antibiotic doses during face-to-face interviews.

## Results

### Patient’s demographic and disease information

The study encompassed 405 pediatric patients, categorized by age, sex, and patient category according to the type of care they received (outpatient, inpatient, and emergency). The majority were term neonatal (34.3%) and male (56.8%). Samples were evenly distributed among inpatient (33.6%), outpatient (33.3%), and emergency (33.1%) care units. Out of 545 prescribed antibiotics, 57.8% were oral liquid, 36.0% parenteral, and 6.2% oral solid forms. The predominant clinical complications, defined as adverse medical conditions or negative outcomes occurring during the course of a disease, included various fevers (16.3%), pneumonia (12.6%), cough (11.9%), sore throat (10.4%), and nasopharyngitis (9.9%). Detailed information is presented in Table 1.

**Table 1:** Participated patients’ demographics and ^1^clinical complications.

	Frequency	Percentage
**Age category**	(N = 405)	
Term Neonatal	139	34.3
Infancy	81	20.0
Toddler	50	12.3
Early childhood	70	17.3
Middle childhood	49	12.1
Adolescence	16	4.0
**Gender**	(N = 405)	
Female	175	43.2
Male	230	56.8
**Patient’s type**	(N = 405)	
Inpatient	136	33.6
Outpatient	135	33.3
Emergency	134	33.1
**Antibiotic’s dosage form**	(N = 545)	
Oral solid	34	6.2
Oral liquid	315	57.8
Parenteral	196	36.0
**Most frequent clinical complications**^1^ (ICD-11)*	(N = 545)	
Fever (MG26)	85	16.3
Pneumonia (CA40.Z)	66	12.6
Cough (MD12)	62	11.9
Sore throat (MD36.0)	54	10.4
Nasopharyngitis (CA00)	51	9.9
Vomiting (MD90.1)	23	4.4
Diarrhea (ME05.1)	21	4.0
Sepsis (1G40)	16	3.0
Nasal congestion (MD11.9)	13	2.5
Meningitis (1D01.Z)	13	2.5
Allergy (4A8Z)	10	2.0
Fecal abnormalities (ME0Y)	8	1.5

*Several individuals had multiple clinical complications

^1^Clinical complications refer to the outcomes of any disease.

### Frequency analysis of prescribed antibiotics

**Fig 2:**
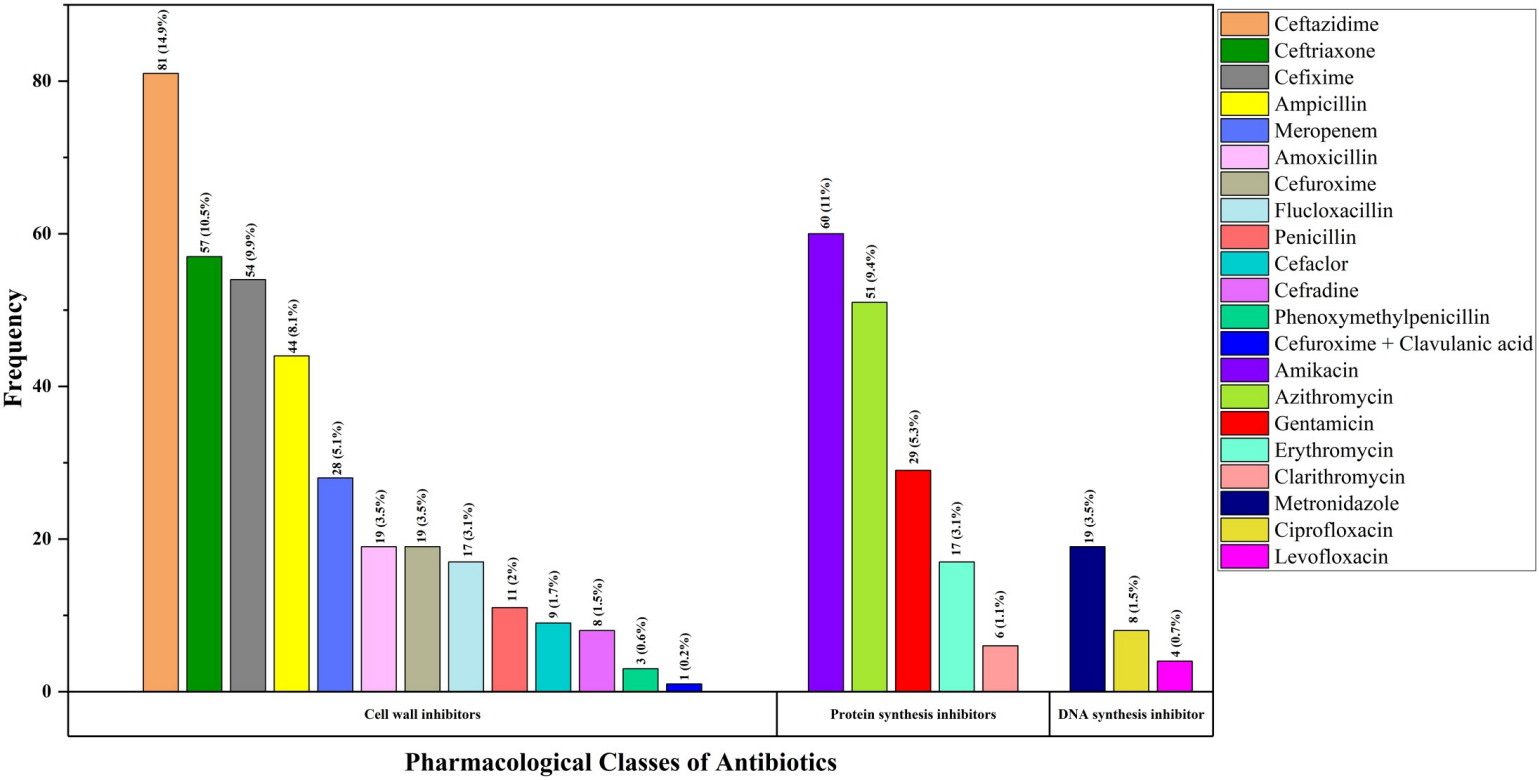
Bar chart showing prescribing percentages of different pharmacological classes of antibiotics in pediatric patients.

Fig 2 below contains the frequency and percentage of different antibiotics, 545 in total prescribed by the physicians to the 405 inpatients, outpatients, and emergency patients who participated in this study. The data revealed that Ceftazidime was the most frequently prescribed antibiotic, with 81 doses (14.9%), followed by Amikacin, with 60 doses (11.0%); Ceftriaxone, with 57 doses (10.5%); Cefixime, with 54 doses (9.9%); and Azithromycin, with 51 doses (9.4%).

### Prevalence of inappropriate antibiotic doses

About 56.8% of pediatric patients (230 out of 405) had inappropriate antibiotic doses (Table 2). Specifically, 61.5% (335 out of 545) of the antibiotics were provided at inappropriate levels. Further analysis revealed that 67.2% (n = 178) of prescriptions had one inappropriate dose, and 37.1% (n = 52) had two inappropriate antibiotic doses. When it came to the various kinds of dosage forms, 80.1% of parenteral antibiotics, 44.4% of oral liquids, and 36.0% of oral solids were prescribed incorrectly.

**Table 2:** Prevalence of inappropriate antibiotic doses.

	Normal		Inappropriate Antibiotic Doses	
	Frequency	Percentage	Frequency	Percentage
**Prevalence of prescriptions**				
Total prescriptions	175	43.2	230	56.8
**Prevalence of antibiotics**				
Prescribed total antibiotics	210	38.5	335	61.5
**Antibiotics per prescription**				
One	87	32.8	178	67.2
Two	88	62.9	52	37.1
**Patient type**				
Inpatient	90	66.2	46	33.8
Outpatient	18	13.3	117	86.7
Emergency	67	50.0	67	50.0
**Dosage form**				
Oral solid	22	64.0	12	36.0
Oral liquid	175	55.6	140	44.4
Parenteral	39	19.9	157	80.1
^1^ **Clinical complications (ICD-11)**				
Fever (MG26)	23	31.9	49	68.1
Pneumonia (CA40.Z)	25	37.9	41	62.1
Cough (MD12)	21	36.2	37	63.8
Sore throat (MD36.0)	36	66.7	18	33.3
Nasopharyngitis (CA00)	26	51.0	25	49.0
Others	44	42.3	60	57.7

^1^Clinical complications refer to the outcomes of any disease.

### Identifying risk factors of antibiotics at inappropriate doses

The study findings highlighted significant predictors of inappropriate antibiotic doses, as revealed through binary univariate and multivariate logistic regression analyses. The multivariate logistic regression analysis underscored that age with a p-value ranging from < 0.001 to 0.032 across categories, type of patient with a p-value < 0.001 to 0.004 across categories, number of prescribed antibiotics with a p-value of 0.026 to 0.041 across categories, and dosage form with p-value ranging from 0.013 to 0.032 across categories exhibited significant associations with inappropriate antibiotic doses, as indicated by adjusted odds ratios (Table 3). Notably, the crude odds ratios, providing insights before adjustment, were initially observed to be different from the adjusted values, emphasizing the impact of potential confounding variables on the associations identified. The regression analysis yielded a statistically significant result (χ^2^ = 135.13, p<0.001), complemented by an R^2^ Cox & Snell value of 0.245. This substantial R^2^ value underscores the model’s effectiveness in explaining variability and strengthens its overall reliability. The likelihood of inappropriate antibiotic doses was markedly lower for infants, toddlers, and early childhood patients compared to term neonates with adjusted odds ratio 0.37, 0.25 and 0.13 respectively. Outpatients and emergency patients had lower odds than indoor patients, having adjusted odds ratio of 0.08, and 0.42, and those prescribed two antibiotics had significantly lower odds than those with a single prescription with 0.45 adjusted odds ratio. Additionally, the parenteral dosage form exhibited a reduced likelihood of inappropriate antibiotic doses compared to oral liquid and solid dosage forms with 0.20 adjusted odds ratio.

**Table 3:** Identification risk factors of antibiotics at inappropriate doses.

	Crude OR	95% CI	*p* value	Adjusted OR	95% CI	*p* value
**Age category**						
Term neonatal	Ref.			Ref.		
Infancy	0.42	0.189–0.932	0.032*	0.37	0.161–0.864	0.021*
Toddler	0.29	0.116–0.725	0.009*	0.25	0.096–0.653	0.005*
Early childhood	0.15	0.059–0.396	<0.001*	0.13	0.051–0.352	<0.001*
Middle childhood	0.31	0.074–1.308	0.112	0.27	0.063–1.176	0.081
Adolescence	0.98	0.148–6.463	0.97	1.04	0.161–6.728	0.967
**Weight**						
<10 kg	Ref.			Ref.		
10–19 kg	0.75	0.287–1.962	0.547	0.68	0.246–1.871	0.454
>19 kg	0.58	0.128–2.637	0.501	0.52	0.111–2.421	0.404
**Patient type**						
Inpatient	Ref.			Ref.		
Outpatient	0.1	0.042–0.236	<0.001*	0.08	0.036–0.194	<0.001*
Emergency	0.47	0.271–0.816	0.004*	0.42	0.245–0.735	0.002*
**Number of prescribed antibiotics**						
One	Ref.			Ref.		
Two	0.52	0.256–1.052	0.041*	0.45	0.222–0.906	0.026*
**Dosage Regimen**						
3 or less days	Ref.			Ref.		
4 to 6 days	0.63	0.306–1.304	0.19	0.58	0.282–1.203	0.144
7 or more days	1.28	0.586–2.803	0.534	1.44	0.674–3.06	0.348
**Dosage form**						
Oral solid	Ref.			Ref.		
Oral liquid	0.35	0.107–1.144	0.081	0.30	0.089–1.031	0.056
Parenteral	0.25	0.068–0.906	0.032*	0.20	0.055–0.714	0.013*

*Statistically significant with p-value less than or equal to 0.05 (95% CI)

### Association between level of doses of antibiotics and patient’s age category, patient type, dosage form, and dosage regimen

Table 4 contains the cross-tabulated frequencies and percentages between the level of doses of antibiotics and the patient’s age, patient type, dosage form and dosage regimen. The Pearson Chi-square test indicated a significant statistical association between doses level of antibiotics and patient type with test statistic 81.472 (*df* = 4, *P* < .001), dosage form with test statistic 51.657 (*df* = 4, *P* < .001), dosage regimen with test statistic 17.019 (*df* = 4, *P* = .002) and, age category with test statistic 77.225 (*df* = 10, *P* < .001) (S1 Table).

**Table 4:** Cross tabulation of the level of doses of antibiotics and patient’s age category, patient type, dosage form, and dosage regimen.

	Level of Doses					
	Normal		Underdose		Overdose	
	Frequency	Percentage	Frequency	Percentage	Frequency	Percentage
**Patient type**	***p*<0.001**					
Inpatient	90	66.2	19	14.0	27	19.9
Outpatient	18	13.3	41	30.4	76	56.3
Emergency	67	50.0	22	16.4	45	33.6
**Dosage form**	***p*<0.001**					
Oral solid	22	64.7	4	11.8	8	23.5
Oral liquid	175	55.6	53	16.8	87	27.6
Parenteral	39	19.9	54	27.6	103	52.6
**Dosage regimen**	***p = 0*.002**					
3 or less days	63	57.3	15	13.6	32	29.1
4 to 6 days	70	43.8	43	26.9	47	29.4
7 or more days	102	37.1	54	19.6	119	43.3
**Age category**	***p*<0.001**					
Term Neonatal	93	66.9	23	16.5	23	16.5
Infancy	27	33.3	17	21.0	37	45.7
Toddler	13	26.0	17	34.0	20	40.0
Early childhood	12	17.1	11	15.7	47	67.1
Middle childhood	21	42.9	11	22.4	17	34.7
Adolescence	9	56.3	3	18.8	4	25.0

The cross-tabulation reveals deviations from normal antibiotic dosage levels across various categories. Among outpatients, most received incorrect antibiotic dosages, with a significant portion being prescribed an overdose. Parenteral forms followed a similar trend. Longer dosage regimens often result in overdoses. Infants and toddlers frequently received incorrect doses, with early childhood patients particularly prone to overdosing. Table 4 provides a comprehensive overview of these patterns.

### Association between frequency of doses of antibiotics and patient’s age category, and patient type

The Pearson Chi-square test, conducted at a 0.05 significance level, assessed the relationship between daily antibiotic consumption frequency and patient characteristics (Table 5).

**Table 5:** Cross tabulation of frequency of doses of antibiotics and patient’s age category, and patient type.

	Frequency of Doses					
	Less frequent		Standard		More frequent	
	Frequency	Percentage	Frequency	Percentage	Frequency	Percentage
**Patient type**	***p*<0.001**					
Inpatient	38	27.9	92	67.6	6	4.4
Outpatient	46	34.1	78	57.8	11	8.1
Emergency	10	7.5	119	88.8	5	3.7
**Age category**	***p* = 0.002**					
Term Neonatal	40	28.8	94	67.6	5	3.6
Infancy	20	24.7	49	60.5	12	14.8
Toddler	13	26.0	35	70.0	2	4.0
Early childhood	9	12.9	59	84.3	2	2.9
Middle childhood	8	16.3	40	81.6	1	2.0
Adolescence	4	25.0	12	75.0	0	0.0

Significant associations were found with patient type (χ^2^ = 34.769, df = 4, p < 0.001) and patient age category (χ2 = 27.312, df = 10, p = 0.002) (S2 Table). Table 5 displayed the cross-tabulated frequencies and percentages. Emergency patients had a higher standard dosage frequency (88.8%) compared to outdoor patients (57.8%). Among pediatric age categories, varying percentages received antibiotics with standard frequency: term neonates (67.6%), infants (60.5%), toddlers (70%), early childhood (84.3%), middle childhood (81.6%), and adolescents (75%).

### Median deviation of antibiotic dose from standard dose level

**Fig 3:**
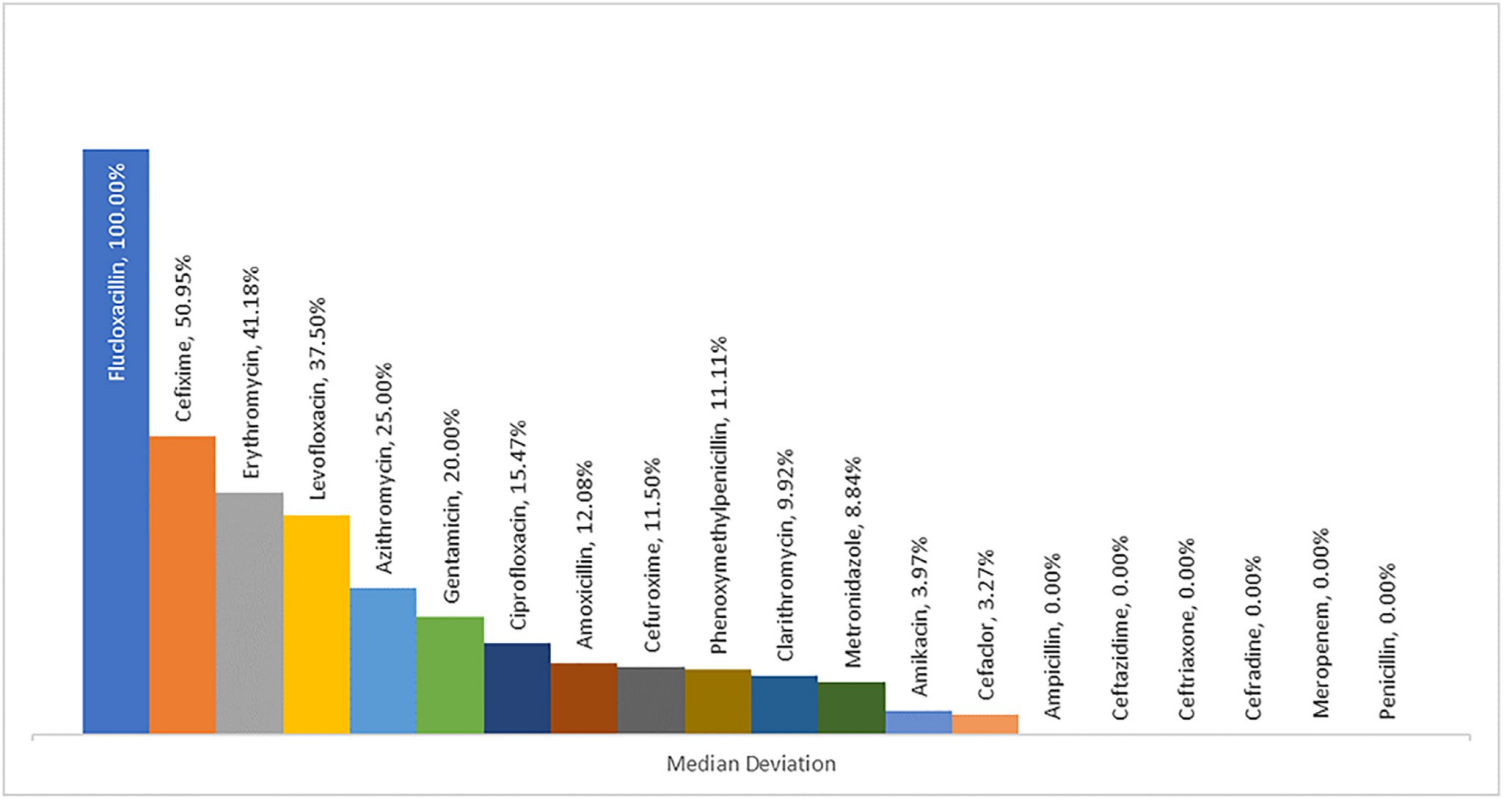
Median deviation of antibiotic dose from standard dose level.

Fig 3 illustrated the median dose deviations for various antibiotics; notably, Flucloxacillin, exhibited a 100% dose deviation from the standard level, whereas, Cefexime showed a lesser dose deviation of 50.95%. Conversely, Ampicillin, Ceftazidime, Ceftriaxone, Meropenem, and others showed 0% median dose deviations from the standard.

### Test of deviation of dose from the standard dose amount concerning patient type and age category

The median absolute deviation from the standard antibiotic dose was 0% for inpatients, 25% for outpatients, and 31.19% for emergency patients. The Kruskal-Wallis test showed significant differences in deviation by patient type (test statistic 62.55, df = 2, p < 0.001), with outpatients and emergency patients experienced more dose deviation from the standard dose than inpatients.

The median absolute deviation of dose by age category was 0% for neonatal patients, 7.63% for toddlers, and 11.11% for middle childhood. Early childhood experienced a dose deviation of 33.93%, infants 37.08%, and adolescents 50%. Thus, early childhood, infancy, and adolescence experienced higher dose deviations from the standard dose compared to neonates and toddlers. The Kruskal-Wallis test (χ2 = 62.721, df = 5, p < 0.001) indicated significant differences in median absolute dose deviations across age categories (S3 Table).

### Prescribed antibiotic doses with respect to the most frequent clinical complications

Table 6 shows that the level of doses prescribed were mostly overdose for diarrhea (52%), vomiting (52%), fever (43%), pneumonia (44%), allergy (40%), and cough (38%). The level of doses prescribed was mostly normal or standard for fecal abnormalities (63%), sore throat (67%), meningitis (54%), nasal congestion (54%), nasopharyngitis (51%), sepsis (50%), and allergy (40%).

**Table 6:** Crosstabulation of most frequent clinical complications and level of antibiotic doses.

	Level of Doses		
	Normal	Underdose	Overdose
**Clinical complications (ICD-11)**	**n (%)**	**n (%)**	**n (%)**
Fever (MG26)	23 (32%)	18 (25%)	31 (43%)
Pneumonia (CA40.Z)	25 (38%)	12 (18%)	29 (44%)
Cough (MD12)	21 (36%)	15 (26%)	22 (38%)
Sore throat (MD36.0)	36 (67%)	8 (15%)	10 (19%)
Nasopharyngitis (CA00)	26 (51%)	10 (20%)	15 (29%)
Vomiting (MD90.1)	7 (30%)	4 (17%)	12 (52%)
Diarrhea (ME05.1)	6 (29%)	4 (19%)	11 (52%)
Sepsis (1G40)	8 (50%)	3 (19%)	5 (31%)
Nasal congestion (MD11.9)	7 (54%)	3 (23%)	3 (23%)
Meningitis (1D01.Z)	7 (54%)	2 (15%)	4 (31%)
Allergy (4A8Z)	4 (40%)	2 (20%)	4 (40%)
Fecal abnormalities (ME0Y)	5 (63%)	1 (13%)	2 (25%)

## Discussion

Best known to us, this study was the first to report a total of 61.5% inappropriate antibiotic doses in pediatric patients in Bangladesh. The findings of the study highlighted the concerning fact that a significant portion of the antibiotic dosages administered to pediatric patients could induce undesirable outcomes. These errors in dosing were significant, as they had the potential to lead to adverse events or the failure of the treatment plan. It is crucial to understand that improving prescription processes and ensuring precise doses are not just procedural enhancements, but essential steps that significantly improve patient safety and the efficacy of the treatment. These actions are essential in our pursuit of improved healthcare outcomes. The results of this investigation are consistent with an earlier study conducted among Malaysian pediatric outpatients, where the reported wrong antibiotic dosing was 64.1% [39]. However, comparatively much lower percentages of wrong dosage were observed in three other studies, where the reported pediatric antibiotic dosing errors were 2.67%, 34%, and 51.3% respectively [9, 12, 40]. The difference in findings between our study and other related studies indicated that healthcare facilities in these countries may possibly lack antibiotic dosing standardization for pediatrics.

As to the occurrence of errors in prescriptions, the present study has observed 56.8% of pediatric prescriptions with inappropriate antibiotic dosing. The study found that a substantial number of prescriptions contained incorrect antibiotic dosages, highlighting a significant gap between theoretical knowledge and practical application among healthcare professionals. This discrepancy indicates that prescribers may not be effectively applying their theoretical knowledge to practical prescribing situations, resulting in inaccuracies in antibiotic dosage. Inappropriate antibiotic prescriptions were also found in hospital emergency departments in two distinct studies. One of the studies revealed a rate of 51.9% for such prescriptions [41], whereas the other study observed a considerably lower rate of 22.9% [42]. Also, while comparing our results to those of two other unique studies carried out in pediatric inpatient care settings in other nations, the former found that the rates of inappropriate antibiotic prescriptions were lower, at 40.8% and 36.5%, respectively [11, 34].

The present research also revealed that this highly prevalent inappropriate antibiotic dosing was found among 33.8% (46 out of 136) pediatric inpatients, 86.7% (117 out of 135) pediatric outpatients and 50.0% (67 out of 134) pediatric emergency patients. The investigation revealed that a significant proportion of prescriptions failed to comply with the specified pediatric dosage guidelines. A comprehensive approach must be taken to improve dosage accuracy and assure the safety of pediatric patients, considering the widespread prevalence of this problem.

When it comes to the incorrect prescribing of antibiotics, there has been a noticeable discrepancy between our study and studies conducted in other countries. Indeed, prior research suggested that our study exhibited a comparatively higher incidence of inappropriate antibiotic prescriptions in comparison to other investigations. However, the results of this study are really worrisome, since the prevalence of inappropriate antibiotic prescriptions constitutes a main issue for the incidence of drug resistant microorganisms. Prescribing antibiotics with incorrect frequency and miscalculated doses may increase the likelihood of antibiotic resistance and treatment failure, according to research published in 2019 [11]. Therefore, the high prevalence rates of inappropriate antibiotic prescriptions indicated the unfamiliarity of prescribers regarding the proper dose calculation, age- and weight-appropriate antibiotic doses, and the necessity of performing additional manipulation considering the pharmacokinetic disparities of these pediatric patients.

We also attempted to look at the clinical situation that was more prone to the possibility of inappropriate antibiotic doses. According to the results of our investigation, pediatric outpatients had the highest prevalence of inappropriate antibiotic dosing. Furthermore, the association of dosing frequency and patient’s age category, and patient type reported that outpatients and infancy had relatively higher rates of administering antibiotic doses at incorrect frequency. These results may pave the way for further investigation to establish how the incorrect frequency of administering antibiotic doses ensue safety concerns among pediatric patients. A study in Pakistan also reported that inappropriate dose and frequency errors were common incidences among pediatric patients because of the paucity of pediatric unit doses and hence pediatric doses were manipulated from available adult unit doses [11].

Indeed, the findings of this research indicated that age, type of patient, number of antibiotics prescribed, and dosage form were significantly associated with inappropriate antibiotic use. The odds of prescriptions of inappropriate antibiotic doses were lower for infants, toddlers, and patients in early childhood compared to term neonates. Outdoor and emergency patients were less likely to receive inappropriate antibiotic prescriptions compared to indoor patients. The likelihood of the incidences of inappropriate antibiotic prescriptions was also lower for patients prescribed with two antibiotics compared to one, and for parenteral dosage form compared to oral solid dosage form. The research outcome highlighted the importance of age, patient type, antibiotic number, and dosage form when prescribing antibiotics. Considering these characteristics enabled healthcare providers to prescribe antibiotics more precisely, hence decreasing the likelihood of incorrect dosage and dealing with antibiotic resistance. The evidence underscored the importance of targeted efforts to improve prescribing patterns and also to ensure the effectiveness of antibiotic treatments. The lower odds of prescribing two antibiotics at inappropriate doses might be due to the possibility that the prescribers could treat their patients based on their previous successful therapeutic outcomes using those antibiotics. However, future investigations would further clarify and confirm the significant association between prescribing multiple antibiotics and inappropriate doses prescriptions.

Since the pediatric population is already known to be heterogeneous, existing results underscore the importance of prescribing multiple dosage forms. For this reason, prescribers prescribed parenteral, liquid, and solid dosage forms of various antibiotics, addressing a wide range of pediatric populations. According to our research, when prescribing antibiotics for distinct pediatric categories, the majority of prescribers favored liquid formulations over solid dosage forms. Additionally, the results of the study showed that pediatric patients who received liquid dosage forms of antibiotics were more likely to get inappropriate antibiotic doses. The study results highlighted that parents or legal guardians of pediatric patients often prefer liquid formulations due to their convenience and to avoid challenges associated with swallowing in children. However, this preference may lead to inappropriate antibiotic dosing. Prioritizing education for healthcare professionals and parents or legal guardians of pediatric patients regarding the significance of appropriate dosing is crucial in order to enhance pediatric health outcomes and mitigate the potential health hazards associated with improper antibiotic utilization. In a study, it was argued that liquid preparations were more advantageous because of their high degree of dosage flexibility and convenient administration, making them suitable for a wide range of ages, including infants [43]. Potential factors contributing to inappropriate antibiotic dosing in pediatrics include insufficient communication between prescribers and caregivers of minors, inadequate guidance on adjusting pediatric doses, time limitations for conducting comprehensive assessments to standardize individual doses, and a lack of understanding regarding pediatric patient factors.

In terms of using antibiotics for the recommended durations, the current data clearly showed that high-dose antibiotics were recommended for durations of ≥ 7days and followed by 4 to 6 days. This research also showed that prescribed antibiotics of under doses were recommended for durations of 4 to 6 days, followed by ≥ 7 days and ≤ 3 days, respectively. This study clearly showed a trend of decreasing the average level of antibiotic doses with the increasing dosage regimen. This observed prescribing pattern suggested the need for a strategic approach to prescribing antibiotics, with the goal of using the minimum effective dose for the shortest possible duration. Moreover, the research supports a transition to individualized therapeutic strategies that are designed to fulfill the distinct requirements of every patient, taking into account specific variables such as age, weight, and the seriousness of medical conditions.

A salient finding in our research exhibited that the rate of parenteral antibiotic injection use is 36%, exceeding the WHO standard of 13.4%–24.1% [44]. The aforementioned observations demonstrated a possible discrepancy in health care practices that may require intervention to align them with global standards. The observed population’s excessive reliance on injectable antibiotics may increase their vulnerability to complications that are commonly associated with the use of injections in pediatrics. However, a much lower prescribing rate of 21.1% parenteral injection was reported among pediatric patients in Sierra Leone [45]. On the other hand, the high rates of prescribing parenteral antibiotics were reported 98% in Pakistan and 95.5% in Mozambique [34, 46]. Parenteral administration accounted for 80% to 86% of antibiotics administered to pediatric inpatients at a tertiary hospital in Nigeria, according to an analysis of antibiotic utilization trends [47]. The possible reason behind the differences in these findings may be due to the age variety in pediatric patients, differences in clinical complications encountered, and study differences.

According to this study, early childhood patients received higher doses of antibiotics than patients in other age groups. Patients at toddler age had the highest percentage of under level of any antibiotic doses. It is clearly understood that while manipulating the commercially available antibiotic doses, prescribers might overlook the doses of antibiotics and be unable to adjust the correct dose for patients in their early childhood and toddler ages. Prescribers who were unfamiliar with the proper pediatric guidelines could have a higher risk of committing such medication dosing errors over and over again. However, appropriate or correct doses of antibiotics could ensure safety and successful therapeutic outcomes in pediatric patients.

The higher administering percentages of inappropriate doses of antibiotics may affect the health of the pediatric population by enabling the development of antibiotic resistance or therapeutic failures or certain antibiotic-induced adverse effects. The Daily Star, a prominent Bangladeshi newspaper, was cited in in a research under the headline "Antibiotics use, sale: who needs a prescription?" on January 13, 2020. The study examined the improper utilization of antibiotics and revealed that ceftriaxone, a medication associated with a watch group, was the most commonly used antibiotic among Bangladeshis [48]. This research work also reported the most used 3^rd^ generation cephalosporin named ceftazidime and ceftriaxone among pediatric patients. Previous successes and efficacy against a wide range of bacteria could justify the use of higher-generation antibiotics. However, unnecessary antibiotics and antibiotics at inappropriate doses can also trigger hazardous consequences among pediatric patients.

Regarding the most used antibiotics, the present research reported that the most commonly prescribed antibiotics for pediatric patients were ceftazidime (14.9%), amikacin (11%), ceftriaxone (10.5%), cefixime (9.9%), azithromycin (9.4%), ampicillin (8.1%), Gentamicin (5.3%), and penicillin (2%). As indicated by the study’s findings, numerous kinds of antibiotics were suggested, each recognized for its unique spectrum of activities. When treating pediatric patients, it is necessary for healthcare providers to choose antibiotics that are well-tolerated and have a safe profile.. A multi-center point prevalence assessment of pediatric inpatients revealed that a higher percentage of pediatric patients were prescribed gentamicin (76.5%) and ceftriaxone (80.5%) [49]. However, a separate study conducted on the same category of pediatric patients found that the antibiotics most frequently prescribed were ampicillin (19.5%), gentamicin (14.5%), and ceftriaxone (12.8%) [5]. Fortunately, the prescribing rates of these antibiotics are more satisfying and interestingly lower than those recommended by WHO. It is essential to consider the health of the pediatric patient, despite the fact that minimizing the use of antibiotics can contribute to the decrease of antibiotic resistance (ABR). According to WHO, the recommended frequency of antibiotic use in hospitals should be 20.0–26.8% [50]. These lower percentages of prescriptions of antibiotics may be due to the heterogeneous age ranges of pediatric patients and comparatively less severe clinical complications, financial constraint and so others. Notably, among all the prescribed antibiotics, flucloxacillin has the highest, or all the prescribed flucloxacillin for pediatric patients has shown a median deviation from the standard. However, there were other certain prescribed antibiotics for pediatric patients like ampicillin, ceftazidime, ceftriaxone, meropenem, penicillin, etc. have no median deviation from the standard dose levels. The observed variation in antibiotic doses of pediatric patients could be attributed to various factors, including age, weight, and physiological differences among pediatric patients, which could make it more challenging to accurately calculate the appropriate doses. To ensure the safety of pediatric patients, the antibiotic dose must be carefully adjusted for each individual patient. In contrast, there was a high prevalence of standard frequencies for several antibiotics among patients in the emergency care unit and early childhood. The findings demonstrated that recognized healthcare standards were being followed to successfully handle emergency and critical health conditions. However, patients in the outpatient care unit and those in infancy were shown to have decreased maintenance of recommended frequencies for various antibiotics. The prudent course of action is likely attributable to the less critical conditions addressed in outpatient care and the delicate health considerations required during infancy. The health implications of these results underscore the need for tailored antibiotic use programs that consider the patient’s geographic location, age, and disease severity to optimize treatment efficacy while minimizing the probability of antibiotic-related risks. Generally, a child’s waking day is as little as 12h which is considered much shorter than that of an adult [51]. So pediatric patients’ sleeping hours will be affected if they are recommended more frequent antibiotic doses. Inappropriate antibiotic doses may pose a threat to pediatric patients in the emergency care unit. For this reason, assessing the most appropriate interval is considered one of the prime factors to be needed to evaluate when selecting a drug dosage regimen for a pediatric patient [51].

In this modern medical health system facility, improper dosing for the pediatric patient population is one of the most common medication errors [52, 53]. Inconsistencies in growth rates among pediatric patients of different ages also may act as a prime factor for arising confusion among prescribers to quickly recognize when a dose of antibiotic is incorrect or inappropriate even in patients of a similar age. In emergency patient care, limited available patient information, disease complexity, higher patient turnover, and lack of knowledge of appropriate pediatric drug references can act as significant barriers to select proper doses of antibiotics for pediatric patients. Without knowing pediatric patients’ weight, prescribers cannot select or properly evaluate any appropriate antibiotic doses.

An important drawback attributed to this study is that this study is unable to report any clinical outcome due to inappropriate antibiotic doses in pediatric patients. The major strength of this research work is its exploration to identify antibiotic use at inappropriate doses among heterogeneous pediatric patients of three care units in Bangladesh. This study has become a pioneer in Bangladesh in terms of reporting the prevalence percentages of inappropriate antibiotic doses in pediatrics. The future endeavor is to emphasize investigating and identifying inappropriate antibiotic use among different categorical pediatric patients in Bangladesh as well as ensuring safe and effective use of appropriate antibiotics in all categorical pediatric patients. Another future endeavor will be to explore the hazardous impacts on pediatric patients’ health and development due to the consumption of several unnecessary antibiotics at inappropriate doses.

## Conclusion

The present study concluded that inappropriate antibiotic doses among in-patient, out-patient, and emergency care units were highly prevalent. Many pediatric patients having dosage regimen of longer periods were more likely to experience prescribing antibiotics at inappropriate doses. Therefore, only precise dose measurement while performing any additional dose calculations and manipulations on commercially available antibiotic doses can reduce the incidence of pediatric patients prescribing inappropriate antibiotic doses. The findings of our research are very worrisome for any pediatric patient who needs specialized care, considering their distinctive physiological and developmental characteristics. It is important that prescribers strictly adhere to the pediatric dosing guidelines of antibiotics during prescribing, adverse drug reactions and therapeutic failures will be extremely common among pediatric patients. The study emphasized the potential advantages of including clinical pharmacists in the process of making treatment decisions for pediatric patients. However, the current research did not establish a direct causal relationship between their involvement and a decrease in the prevalence of inappropriate antibiotic dosing. The evidence indicates that clinical pharmacists have the potential to significantly improve prescription accuracy and advance effective clinical pharmacy practices in pediatric care. However, further research is required to confirm their impact on reducing dose errors and improving patient outcomes.

## Supporting information

S1 Table This table displays the significance of the association between the levels of antibiotic doses and pediatric patient-related factors (age category, type, dosage form, and dosage regimen). (DOCX)

S2 Table This table displays the significance of the association between the frequency of antibiotic doses and pediatric patient-related factors (age category, and type). (DOCX)

S3 Table This table presents the results of tests assessing deviations of the administered antibiotic dose from the standard dose amount. The standard dose refers to the recommended amount of medication as per guidelines or protocols. The table evaluates whether the doses given to pediatric patients differ significantly from the standard doses.
(DOCX)

S1 File This file contains the Bangla (Native language) version of the study questionnaire. The questionnaire comprises of two sections: Demographic profile, and Medication history.
(DOCX)

S2 File This file contains the English version of the study questionnaire. The questionnaire comprises of two sections: Demographic profile, and Medication history.
(DOCX)

S1 Data The dataset was coded unanimously and all personal information was excluded during coding the data. This dataset was used for statistical analysis.
(XLSX)
