# Supplementary material for: Prevalence of inappropriate antibiotic doses among pediatric patients of inpatient, outpatient, and emergency care units in Bangladesh: A cross-sectional study
Source: PLOS Glob Public Health. 2024 Sep 10;4(9):e0003657. doi: 10.1371/journal.pgph.0003657 (PMC11386430; doi:10.1371/journal.pgph.0003657)
Supplement: S1 Table — (DOCX) [file pgph.0003657.s001.docx]

**S1 Table. Significance of association between level of doses of antibiotics and patient’s age category, patient type, dosage form and, regimen of doses**

**Table A: Association between patient type and doses level of antibiotics**

|  | Value | df | Asymptotic Significance (2-sided) | Exact Significance (2-sided) |
| --- | --- | --- | --- | --- |
| Pearson Chi-Square | 62.435^a^ | 4 | .000 | .000 |
| Likelihood Ratio | 67.614 | 4 | .000 | .000 |
| Fisher's Exact Test | 66.607 |  |  | .000 |
| Linear-by-Linear Association | 8.011^b^ | 1 | .005 | .005 |
| N of Valid Cases | 405 |  |  |  |

**Table B: Association between dosage form and doses level of antibiotics**

|  | Value | df | Asymptotic Significance (2-sided) | Exact Significance (2-sided) |
| --- | --- | --- | --- | --- |
| Pearson Chi-Square | 39.069^a^ | 4 | .000 | .000 |
| Likelihood Ratio | 40.959 | 4 | .000 | .000 |
| Fisher's Exact Test | 40.502 |  |  | .000 |
| Linear-by-Linear Association | 28.927^b^ | 1 | .000 | .000 |
| N of Valid Cases | 405 |  |  |  |

**Table C: Association between regimen of doses and doses level of antibiotics**

|  | Value | df | Asymptotic Significance (2-sided) | Exact Significance (2-sided) |
| --- | --- | --- | --- | --- |
| Pearson Chi-Square | 18.822^a^ | 4 | .001 | .001 |
| Likelihood Ratio | 19.236 | 4 | .001 | .001 |
| Fisher's Exact Test | 18.714 |  |  | .001 |
| Linear-by-Linear Association | 11.384^b^ | 1 | .001 | .001 |
| N of Valid Cases | 405 |  |  |  |

**Table D: Association between patient’s age category and doses level of antibiotics**

|  | Value | df | Asymptotic Significance (2-sided) | Exact Significance (2-sided) |
| --- | --- | --- | --- | --- |
| Pearson Chi-Square | 56.989^a^ | 10 | .000 | .^b^ |
| Likelihood Ratio | 58.010 | 10 | .000 | .^b^ |
| Fisher's Exact Test | .^b^ |  |  | .^b^ |
| Linear-by-Linear Association | 26.401^c^ | 1 | .000 | .000 |
| N of Valid Cases | 405 |  |  |  |
