# Supplementary material for: Prevalence of inappropriate antibiotic doses among pediatric patients of inpatient, outpatient, and emergency care units in Bangladesh: A cross-sectional study
Source: PLOS Glob Public Health. 2024 Sep 10;4(9):e0003657. doi: 10.1371/journal.pgph.0003657 (PMC11386430; doi:10.1371/journal.pgph.0003657)
Supplement: S2 Table — (DOCX) [file pgph.0003657.s002.docx]

**S2 Table. Significance of association between frequency of doses of antibiotics and patient’s age category and, patient type**

**Table A: Association between patient age and doses frequency of antibiotics**

|  | Value | df | Asymptotic Significance (2-sided) | Exact Significance (2-sided) |
| --- | --- | --- | --- | --- |
| Pearson Chi-Square | 21.868^a^ | 10 | .016 | .^b^ |
| Likelihood Ratio | 20.378 | 10 | .026 | .^b^ |
| Fisher's Exact Test | .^b^ |  |  | .^b^ |
| Linear-by-Linear Association | 2.156^c^ | 1 | .142 | .146 |
| N of Valid Cases | 405 |  |  |  |

**Table B: Association between patient type and doses frequency of antibiotics**

|  | Value | df | Asymptotic Significance (2-sided) | Exact Significance (2-sided) |
| --- | --- | --- | --- | --- |
| Pearson Chi-Square | 20.692^a^ | 4 | .000 | .000 |
| Likelihood Ratio | 22.610 | 4 | .000 | .000 |
| Fisher's Exact Test | 22.188 |  |  | .000 |
| Linear-by-Linear Association | 7.364^b^ | 1 | .007 | .007 |
| N of Valid Cases | 405 |  |  |  |
