# Supplementary material for: Prevalence of inappropriate antibiotic doses among pediatric patients of inpatient, outpatient, and emergency care units in Bangladesh: A cross-sectional study
Source: PLOS Glob Public Health. 2024 Sep 10;4(9):e0003657. doi: 10.1371/journal.pgph.0003657 (PMC11386430; doi:10.1371/journal.pgph.0003657)
Supplement: S3 Table — The standard dose refers to the recommended amount of medication as per guidelines or protocols. The table evaluates whether the doses given to pediatric patients differ significantly from the standard doses. (DOCX) [file pgph.0003657.s003.docx]

**S3 Table: Test of deviation of dose from the standard dose amount**

**Table A: Kruskal-Wallis Test for patient type**

| **Ranks** | | | |
| --- | --- | --- | --- |
|  | Patient type | N | Mean Rank |
| percent off err | Indoor | 136 | 107.25 |
|  | Outdoor | 135 | 199.12 |
|  | Emergency | 134 | 145.52 |
|  | Total | 405 |  |

| **Test Statistics^a,b,c^** | |
| --- | --- |
|  | percent off err |
| Kruskal-Wallis H | 62.554 |
| df | 2 |
| Asymptotic Significance | .000 |
| a. Kruskal Wallis Test | |
| b. Grouping Variable: Patient type | |
| c. Some or all exact significances cannot be computed because there is insufficient memory. | |

**Table B: Kruskal-Wallis Test for patient’s age category**

| **Ranks** | | | |
| --- | --- | --- | --- |
|  | Age | N | Mean Rank |
| percent off err | Term Neonatal | 139 | 101.89 |
|  | Infancy | 81 | 155.84 |
|  | Toddler | 50 | 192.19 |
|  | Early childhood | 70 | 199.90 |
|  | Middle childhood | 49 | 160.65 |
|  | Adolescence | 16 | 167.82 |
|  | Total | 405 |  |

| **Test Statistics^a,b^** | |
| --- | --- |
|  | percent off err |
| Kruskal-Wallis H | 62.721 |
| df | 5 |
| Asymptotic Significance | .000 |
| a. Kruskal Wallis Test | |
| b. Grouping Variable: Age | |

**Note:** Here, the Kruskal-Wallis test is performed because in both cases (patients type and age group), the data are nonparametric (not normally distributed) and have categories more than two.
