# Supplementary material for: Prevalence of inappropriate antibiotic doses among pediatric patients of inpatient, outpatient, and emergency care units in Bangladesh: A cross-sectional study
Source: PLOS Glob Public Health. 2024 Sep 10;4(9):e0003657. doi: 10.1371/journal.pgph.0003657 (PMC11386430; doi:10.1371/journal.pgph.0003657)
Supplement: S1 File — The questionnaire comprises of two sections: Demographic profile, and Medication history. (DOCX) [file pgph.0003657.s004.docx]

**Prevalence of inappropriate antibiotic doses among pediatric patients of in-patient, out-patient, and emergency care units in Bangladesh: a cross-sectional study**

* Do you want to participate in our study?

Yes

No

***Note: All of your personal details including name will not disclose anywhere and your privacy will remain confidential***

# **Section 1: Demographic Profile**

1. **Patient’s ID: Name of the patient: …………………………………**
2. **Age:** years/months/days
3. **Gender:** Choose only one option

Male

Female

1. **Weight:** kilogram (kg)

# **Section 2: Medication History**

1. **In which care unit are you staying now?**

Indoor care unit

Outdoor care unit

Emergency care unit

1. **For which disease you are staying in care unit: …………………………………….**
2. **Name of prescribed antibiotics: ……………………………………………………..**
3. **Amount of dose:** milligram (mg)
4. **Frequency of dose:**  hourly
5. **Regimen of dose:** days
6. **Form of dose: …………………………………………………………………………**
