# Supplementary material for: Prevalence of inappropriate antibiotic doses among pediatric patients of inpatient, outpatient, and emergency care units in Bangladesh: A cross-sectional study
Source: PLOS Glob Public Health. 2024 Sep 10;4(9):e0003657. doi: 10.1371/journal.pgph.0003657 (PMC11386430; doi:10.1371/journal.pgph.0003657)
Supplement: S2 File — The questionnaire comprises of two sections: Demographic profile, and Medication history. (DOCX) [file pgph.0003657.s005.docx]

**বাংলাদেশে অন্তর্বিভাগ, বহির্বিভাগ এবং জরুরী চিকিৎসা বিভাগীয় শিশু রোগীদের মধ্যে অনুপযুক্ত এন্টিবায়োটিক ডোজের প্রাদুর্ভাব: একটি ক্রস-বিভাগীয় গবেষণা**

* আপনি কি আমাদের গবেষণায় অংশগ্রহণ করতে চান?

হ্যাঁ

না

**দ্রষ্টব্য: নামসহ আপনার সমস্ত ব্যক্তিগত তথ্য কোথাও প্রকাশ করা হবে না এবং আপনার গোপনীয়তা অবশ্যই রক্ষা করা হবে**

# **বিভাগ – ১: জনতাত্ত্বিক পরিলেখ**

**১। রোগীর শনাক্তকরণ নম্বর: রোগীর নাম: …………………………………**

**২। বয়স:** বছর/মাস/দিন

**৩। লিঙ্গ:** শুধুমাত্র একটি বিকল্প পছন্দ করুন

ছেলে

মেয়ে

**৪। ওজন:** কিলোগ্রাম (কেজি)

# **বিভাগ - ২: চিকিৎসা বিষয়ক তথ্য**

**৫। আপনি এখন হাসপাতালের কোন ইউনিটে আছেন?**

অন্তর্বিভাগ

বহির্বিভাগ

জরুরি চিকিৎসা বিভাগ

**৬। যে রোগের জন্য চিকিৎসা নিচ্ছেন:** …………………………………………………………………………

**৭। নির্দেশিত এন্টিবায়টিকের নাম:** …………………………………………………………

**৮। ডোজের পরিমাণ:** মিলিগ্রাম (মি.গ্রা.)

**৯। ডোজের পুনরাবৃত্তিক হার:**  ঘণ্টা

**১০। ডোজের স্বাস্থ্যবিধান:** দিন

**১১। ডোজের ধরণ:** …………………………………………………………………………
